# MTPA control of permanent magnet synchronous motor based on dual-vector model predictive control

**DOI:** 10.1371/journal.pone.0262135

**Published:** 2022-01-21

**Authors:** Ningzhi Jin, Chao Wang, Dongyang Sun, Zelin Li, Kai Zhou

**Affiliations:** Engineering Research Center of Automotive Electronics Drive Control and System Integration, Ministry of Education, Harbin University of Science and Technology, Harbin, Heilongjiang, China; J.C. Bose University of Science and Technology, YMCA, INDIA, INDIA

## Abstract

The vector control of the permanent magnet synchronous motor (PMSM) is affected by cross-coupling, output delay, parameter mismatch, and other factors; thus, resulting in its poor steady-state and insufficient dynamic performance. To address these problems, the design proposed in this study adopts a model predictive current control strategy. In the traditional model predictive control, the absolute value of the difference between the predicted output current of the inverter and the reference current is used as the cost function instead of the minimum value of the valence function, i.e., the optimal solution of the system, and the best switching state is outputted. The design proposed in this study adopts the dual-vector model to predict the current control. Firstly, the reference voltage vector was predicted on basis of the deadbeat idea, which reduced the calculation burden of processor. Next, in order to further improve the stability of the system, a two-vectors duty cycle calculation method was introduced. Then, simplifies the selection range of the two voltage vectors. While ensuring the accuracy of the voltage vector, the control is reduced. Reduce the amount of calculation in the system, thereby improving its robustness. Finally, based on the principle of current vector tracking error minimization, the duration of the selected voltage vector was determined. Last but not the least, the control strategy is applied to the MTPA control to increase the operating efficiency of the control motor. The improved control strategy can effectively reduce the torque ripple and improve the dynamic and steady-state performance of the system. Simulation results verify the feasibility and effectiveness of the proposed control algorithm.

## 1 Introduction

A permanent magnet synchronous motor (PMSM) has the characteristics of small size, high power density, simple structure, and a large torque-to-inertia ratio [1]. In addition, with the improvement in the performance of rare earth permanent magnet materials, a gradual reduction in their costs, and the development of power electronics technology, PMSMs have attracted the favor of many researchers, and their application fields have been gradually promoted [2].

In the PMSM control system, the performance of the current inner loop is a key factor that affects the performance of the control system. At present, the following methods are being used for controlling the current of a PMSM: Wang et al. [3] have adopted the proportional-integral current control. Its controller has a simple structure and high control accuracy. However, it is limited by bandwidth, exhibits poor dynamic performance, and its parameter tuning is difficult. Jinguo et al. [4] used the hysteresis current control, which is highly robust and has a rapid response. However, it has problems such as nonfixed switching frequency and large current ripple. Other studies [5–8] have used models to predict the current control by directly calculating the parameters obtained by the system feedback using the predictive model and by using the motor equation to obtain the voltage value required to reach a given current value. This method can achieve superior dynamic performance and smaller current fluctuations.

Model predictive control (MPC) is a control method based on the future state of the discrete-time of the object that is being controlled. The MPC system exhibits good dynamic response performance. It uses value functions to find the optimal solution that can achieve multi-objective and nonlinear control [9].

MPC is divided into CCS-MPC and FCS-MPC according to whether the pulse width modulator is needed. Among them, CCS-MPC has a good development prospect in tracking low steady-state ripples. Hammoud et al. [10] mentions the real-time realization of the motor’s offset-free performance.

The finite control set MPC is used for replacing the traditional proportional-integral (PI) current controller in the vector control. The current value is predicted under different switching states in the future, and the optimal value function is selected for the predictive current corresponding to the switching state of the voltage vector acting on the inverter [11]. The limited control set MPC fully considers the discrete characteristics of the inverter and has the advantages of simple control technique, easy handling of multiple constraints, and high dynamic performance.

However, due to its limitations, the traditional finite control set MPC has only one switching state acting on the inverter in a control cycle; thus, resulting in large current fluctuations and poor system steady-state performance. In addition, six enumeration predictions are used, which results in a large computational burden [12]. Hammoud et al. [13] and Zhang et al. [14] proposed methods to improve computational efficiency to overcome this problem, but there are still shortcomings. To achieve improved control performance from the traditional predictive control, its sampling frequency and control frequency must be increased, and the average switching frequency of traditional model predictive control is 10kHz, which requires complex system hardware. At present, the improved algorithm for the limited control set MPC mainly focuses on the improvement of its steady-state performance, which has been developed from the single vector control of the traditional MPC [15, 16] to a multivector control [17, 18].

Jing et al. [19] and Zhang et al. [20] aimed at the problem of poor steady-state performance caused by only one voltage vector in each sampling period of the traditional MPC strategy. They proposed the duty cycle MPC strategy in which duty cycle control is added to the traditional MPC strategy. In this method, the calculation of the empty ratio is performed after the voltage vector is selected. However, the selected voltage vector cannot be guaranteed to be globally optimal.

Liu et al. [21] proposed an optimal duty cycle MPC torque control strategy for asynchronous motors. In this method, the voltage vector and duty cycle were simultaneously added to the value function for optimization, which ensured the global optimization of the selected voltage vector and significantly improved the steady-state performance of the system. However, since the second voltage vector is always a zero-voltage vector, the steady-state achieved using this method at high speed is poor.

Huang et al. [22] aimed at the problem of the second voltage vector always being a zero-voltage vector [21] and proposed a generalized double-vector-based MPC torque control strategy for asynchronous motors, which extends the selection range of the voltage vector to any. In the range of the two voltage vectors, the value function is used for selecting the two voltage vectors simultaneously in the voltage vectors of the pairwise combination. However, due to the optimization of the 49 voltage vector combinations, the amount of computation required is relatively large, and the hardware requirements are relatively high. Thus, the application of this method is considerably difficult.

The abovementioned methods have achieved the goal of improving the steady-state performance of the control system. However, due to the increase in the number of vectors and the diversification of the vector combination methods, the control complexity and the computational load of the algorithm also increase. In order to ensure the realization of other functions in the control system, such as the speed measurement, the current measurement, and the observer algorithms that are usually required for motor control, it is necessary to seek a more concise algorithm to reduce the computational complexity of the algorithm and improve the real-time performance of the system.

Based on the discussion and analysis above, a model predictive current control system with good steady-state performance, strong anti-interference, and better robustness was designed in this study.. The main contributions of this research are as follows:

1. Build the state equation by according to the characteristics of the permanent magnet motor, and then obtain the current prediction formula through the first-order Euler discretization method, which provides the basis for the theoretical analysis of the subsequent model predictive current control.
2. Design a dual-vector MPCC with small calculation amount and good robustness, and design a new voltage vector selection method. The steady-state performance and dynamic anti-interference ability are tested in the designed control system.
3. Propose a method of distribution of voltage vector action time, and obtain the optimal time distribution plan by combining with the value function.

## 2 Conventional MPC of a PMSM

### 2.1 Mathematical model of PMSM and MTPA control

The state equations of the d-axis and q-axis components of the stator current of the PMSM are:

$$\begin{array}{l} \frac{d}{dt}i_{d}=\frac{1}{L_{d}}(u_{d}‐R_{s}i_{d}+\omega_{e}L_{q}i_{q})\\ \frac{d}{dt}i_{q}=\frac{1}{L_{q}}(u_{q}‐R_{s}i_{q}‐\omega_{e}(L_{d}i_{d}+\psi_{f})) \end{array}$$ (1)

where *u*_d_, *u*_*q*_ are the d, q-axis stator voltage components, *i*_*d*_, *i*_*q*_ are the d-, q-axis stator current components, *L*_d_, *L*_q_ are the d-, q-axis inductance components,

*ω*_e_ is the electrical angular velocity, R is the stator resistance, and *ψ*_f_ is the permanent magnet flux.

Using the first-order Euler method to discretize the mathematical model expressed by Eq (1), the stator current prediction model can be obtained.

$$\begin{array}{l} i_{d}(k+1)=i_{d}(k)+\frac{T_{s}}{L_{d}}(u_{d}(k)‐Ri_{d}+\omega_{e}L_{q}i_{q})\\ i_{q}(k+1)=i_{q}(k)+\frac{T_{s}}{L_{q}}(u_{q}(k)‐Ri_{q}‐\omega_{e}(L_{d}i_{d}+\psi_{f})) \end{array}$$ (2)

where *i*_d_(*k*), *i*_q_(*k*) are the d-and q-axis current at the *k*th sampling time, *i*_d_(*k*+1), *i*_q_(*k*+1) are the predicted values of the d-and q-axis current at the (*k+*1)th sampling time, *T*_s_ is the sampling period, *u*_d_(*k*), *u*_q_(*k*) are the *k*th d-and q-axis voltages, *ω*_e_ is the electrical angular velocity and is given by *ω*_e_ = *n*_p_*ω_r_, where *n*_p_ is the number of pole pairs, and *ω*_r_ is the mechanical angular velocity.

The torque equation is given by

$$T_{e}=\frac{3}{2}p_{n}i_{s}(\psi_{f}+(L_{d}‐L_{q})i_{s}*\cos\beta )*\sin\beta$$ (3)

where *β* is the current vector angle, *i*_s_ is the stator current amplitude in the rotating coordinate system, and *L*_d_, *L*_q_ are the equivalent inductance of the quadrature axis. According to the principle of MTPA, when *i*_s_ is constant, the derivative of *β* can be obtained from the equation that meets the relationship between the d-q axis current to satisfy the MTPA curve.


$$i_{d}=\frac{‐\psi_{f}+\sqrt{\psi_{f}^{2}+4{\left(L_{d}‐L_{q}\right)}^{2}i_{q}^{2}}}{2(L_{d}‐L_{q})}$$ (4)

### 2.2 Dual-vector MPC based on MTPA

**Fig 1:**
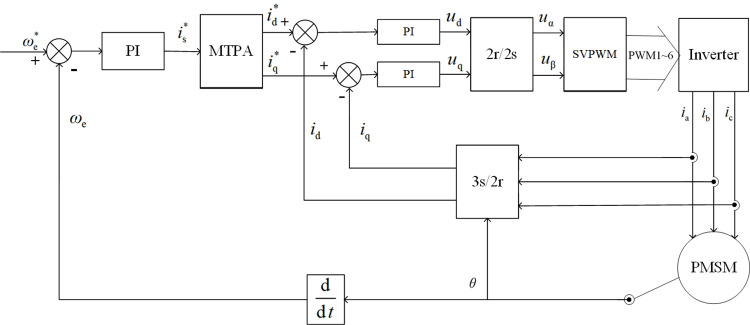
Block diagram of the traditional MTPA control system.

As shown in Fig 1, in the PMSM double closed-loop vector control system of the traditional maximum torque-to-current ratio control strategy, the current inner loop regulator usually adopts a PI control method. When the given torque of the motor or the external load torque has a sudden change, according to the MTPA principle, the given right-angle axis current will also produce a large sudden change, resulting in the current regulator reaching saturation in a short time. As a result, the actual motor stator current trajectory deviates from the MTPA curve and produces a large torque ripple. Taking $\frac{d}{dt}i_{q}=0$, we get,

$$i_{q}=‐\frac{\omega_{e}L_{d}}{R_{s}}i_{d}+\frac{u_{q}‐\omega_{e}\psi_{f}}{R_{s}}$$ (5)

From Eq (5), it can be seen that as the motor runs into the high-speed area, *ω*_e_ of the motor will increase, and the coupling between the currents of the AC and DC shafts will deepen, resulting in a decrease in the system stability. Therefore, the finite set MPCC method has been used in this study to replace the current inner loop in the traditional MTPA control, which improves the stability of the system.

By using the forward Euler formula to discretize Eq (1), the predicted value of the right-angle axis current model of the PMSM at the time *k+*1 can be obtained from that at time *k*. The specific prediction model is as follows:

$$\begin{array}{l} i_{d}(k+1)=i_{d}(k)+\frac{T_{s}}{L_{s}}[u_{d}(k)‐R_{s}i_{d}(k)+\omega_{r}(k)L_{s}i_{q}(k)]\\ i_{d}(k+1)=i_{d}(k)+\frac{T_{s}}{L_{s}}[u_{d}(k)‐R_{s}i_{d}(k)‐\omega_{r}(k)L_{s}i_{q}(k)‐\omega_{r}(k)\psi_{f}] \end{array}$$ (6)

According to the switching principle of the insulated-gate bipolar transistor (IGBT), the inverter can only output six effective voltage vectors and two zero vectors. The finite set MPC makes full use of this discrete characteristic. Its core control idea is as follows: The suboptimal voltage vector is selected to obtain the current value of the quadrature axis that meets the minimum value function at the *k*+1th time to enable the system to quickly and dynamically track the MTPA setting. The specific value function is expressed as follows:

$$CF={\left(i_{d}^{*}‐i_{d}(k+1)\right)}^{2}+{\left(i_{q}^{*}‐i_{q}(k+1)\right)}^{2}$$ (7)

where $i_{d}^{*}$, $i_{d}^{*}$ are the given values of the d-and q-axis components of the stator current obtained from MTPA.

## 3 MTPA based dual-vector MPCC strategy

### 3.1 Traditional MPCC strategy

The core idea of the traditional MPCC strategy is as follows: For every cycle, six effective voltage vectors are substituted into the predictive model to obtain the corresponding current predicted value, and this predicted value is selected using the value function to select the optimal voltage vector, i.e., the minimum value function is obtained. The voltage corresponding to the predicted current value of the value is used as the optimal voltage vector.

**Fig 2:**
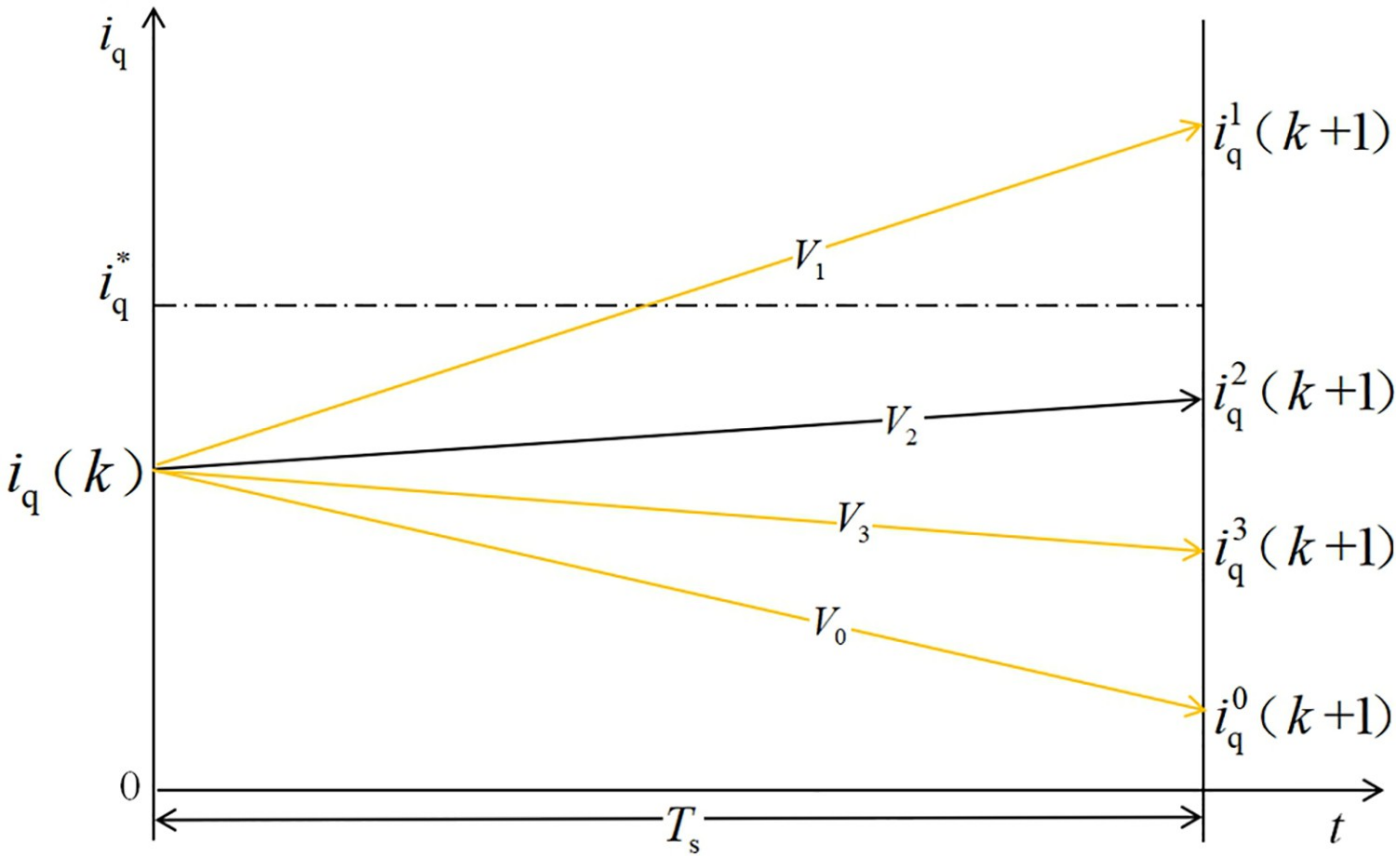
Schematic showing the traditional MPCC vector selection procedure.

It can be seen from Fig 2 that the optimal voltage vector in the traditional MPCC strategy acts for a whole sampling period. When the predicted current value after the optimal voltage vector is greater than or less than the given value, the control quantity will fluctuate greatly, and the system becomes stable. The state performance is poor, and the predicted current is not accurately close to the given current.

### 3.2 Improved dual-vector MPCC strategy

The idea of the dual-vector MPCC strategy is based on the traditional MPCC strategy in which the optimal voltage vector, *V*_opt1_, is elected, and by performing a voltage vector selection, the second optimal voltage vector, *V*_opt2_, is determined. The basis for selecting *V*_opt2_ is as follows: After *V*_opt1_ and *V*_opt2_ work together for a sampling period, the error between the direct and quadrature axis currents, *i*_d_ and *i*_q_, and their given values is the smallest. Thus, the value function, given by Eq (7) can still be used when selecting *V*_opt2_.

#### 3.2.1. Improvement of *V*_opt1_ selection

**Fig 3:**
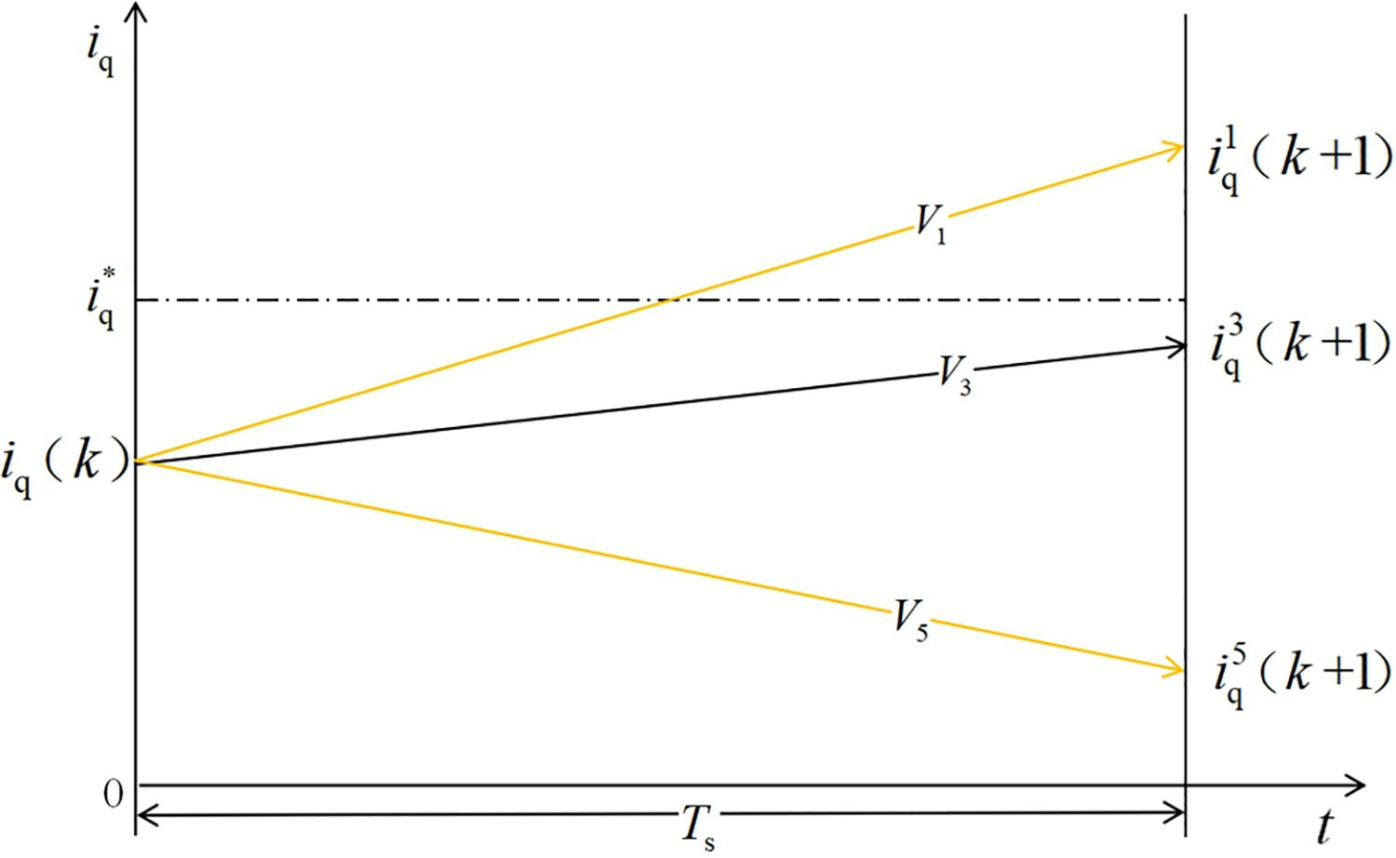
Selection of the first voltage vector.

For the selection of *V*_opt1_, the general dual-vector MPCC adopts the same selection method as the traditional MPCC, i.e., it traverses seven voltage vectors and selects the optimal value using the value function. This design simplifies the selection process and only selects three effective voltages that are 120° apart from each other as the selection range, which can be a combination of *V*_1_, *V*_3_, and *V*_5_ or a combination of *V*_2_, *V*_4_, and *V*_6_. A schematic of the selection procedure is shown in Fig 3.

#### 3.2.2. Introduction to the improved dual-vector MPCC strategy

**Fig 4:**
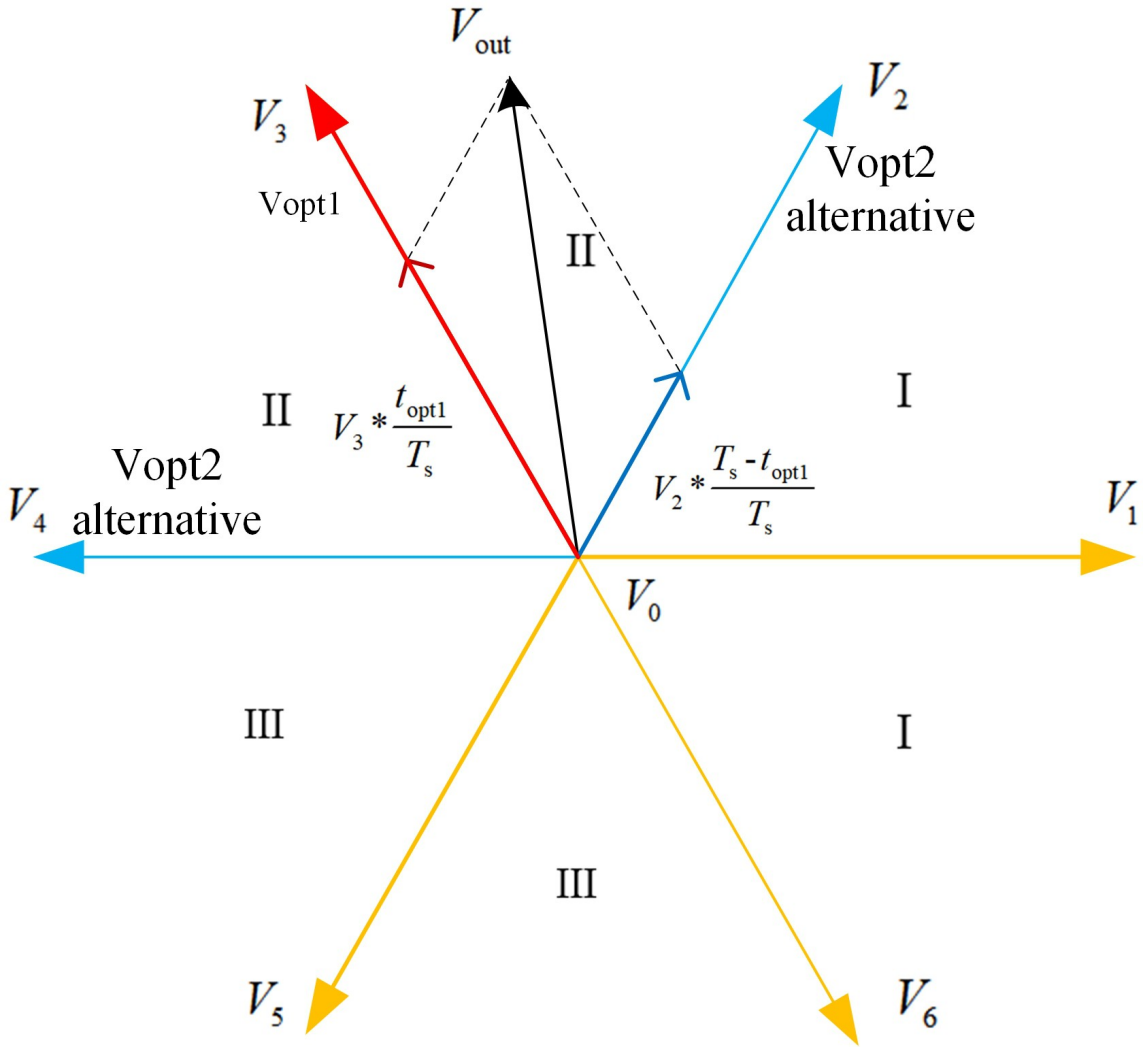
Schematic of the vectors in the voltage vector selection procedure.

As shown in Fig 4, using *V*_1_, *V*_3_, and *V*_5_ as the first voltage vector, the three voltage vectors are divided into three sectors within the limit of ±60°. When the actual voltage vector falls in the first sector, *V*_1_ is used as the first voltage vector, and the adjacent vectors *V*_2_, *V*_6_, and the zero-voltage vector, *V*_0_, are used as alternatives to *V*_opt2_. When the voltage vector falls in sectors II and III, *V*_3_ and *V*_5_ are respectively selected as the voltage vectors.

#### 3.2.3. Improved V_opt2_ selection method

**Fig 5:**
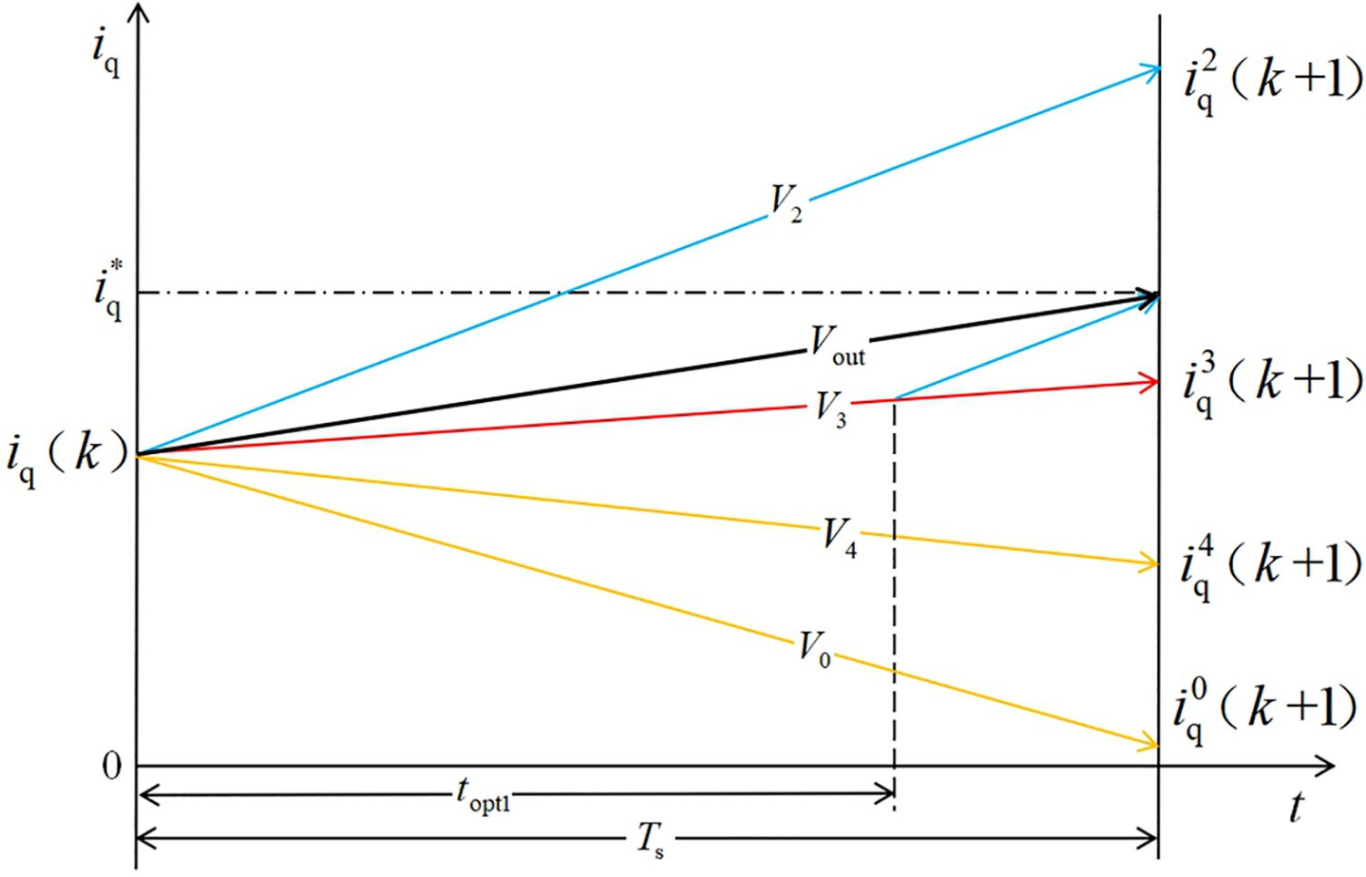
Selection of the second voltage vector.

Fig 5 shows the selection of the second optimal voltage vector, *V*_opt2_. When *V*_opt1_ selects *V*_3_, *V*_opt2_ is selected among *V*_2_, *V*_4_, and *V*_0_ adjacent to *V*_opt1_. Further, the three voltage vectors and *V*_opt1_ are combined separately, and the action times of the two voltages in each voltage combination are preallocated to synthesize a new voltage vector. By comparing the value function, the optimal value of *V*_opt2_ can be obtained by selecting the combination that is closest to the actual voltage.

#### 3.2.4. Calculation of the action time

The combined voltage is calculated as follows:

$$\begin{array}{l} u_{d}{|}_{k=j}=\frac{t_{opt1}}{T_{s}}u_{d\_opt1}+\frac{T_{s}‐t_{opt1}}{T_{s}}u_{dj}\\ u_{q}{|}_{k=j}=\frac{t_{opt1}}{T_{s}}u_{q\_opt1}+\frac{T_{s}‐t_{opt1}}{T_{s}}u_{qj} \end{array}$$ (8)

where *u*_d_|_k = j_, *u*_q_|_k = j_ are the d and q axis components of the voltage vector synthesized by Vopt1 and Vopt2, *u*_d_opt1_, *u*_q_opt1_ are the direct and quadrature axis voltage components of the stator voltage corresponding to *V*_opt1_, *t*_opt1_ is the action time of *V*_opt1_, *u*_dj_, *u*_qj_ are the direct and quadrature axis voltage vectors. Except for *V*_opt1_ and its opposite voltage vector, the stator voltages corresponding to the *j*th voltage vector are the direct and quadrature axis voltage components (*j* = 1,2,3).

Three sets of voltage vector combinations and their action times can provide three current prediction values. By comparing the value function of Eq (7), the voltage vector corresponding to the current prediction value closest to the given current value can be obtained, i.e., For the second optimal voltage vector, the corresponding action time is the optimal action time. Narrowing the selection range of the voltage vector can enable a faster and accurate selection of *V*_opt2_.

On the basis of the traditional MPC optimization via the value function, the dual-vector MPCC strategy also adds the influence of the action time on the selection of the voltage vector, which not only optimizes the selection of the voltage vector but also optimizes the action time. Thus, by preallocating the action time of the voltage vector, the value function selects the optimal combination of the voltage vector and the action time, which can ensure that the final applied voltage vector is still optimal and consequently, the selection of the voltage vector is accurate.

In the case of distributing the action time in the sampling period, the dual-vector MPCC strategy replaces the reference value of the *k*+1th q-axis current in the deadbeat control target with the given q-axis current value at the time *k*, thereby eliminating the current deviation term and avoiding the realization principle of the error caused by the measurement accuracy of the sensor is shown in Figs 3–5.


$$i_{q}(k+1)=i_{q}(k)+s_{opt1}t_{opt1}+s_{j}(T_{s}‐t_{opt1})=i_{q}^{*}$$ (9)

The finishing formula (9) can be obtained, the action time, t_opt1_, of *V*_opt1_ is given by

$$t_{opt1}=\frac{i_{q}^{*}‐i_{q}(k)‐s_{j}T_{s}}{s_{opt1}‐s_{j}}$$ (10)

where *s*_opt1_, *s*_j_ are the slopes of the two voltage vectors, *V*_opt1_ and *V*_j_, and *T*_s_-*t*_opt1_ is the action time. The slopes *s*_opt1_, *s*_j_ can be obtained as follows:

$$\begin{array}{l} s_{\mathrm{opt}l}={\frac{di_{q}}{dt}|}_{u_{q}=u_{q\_\mathrm{opt}1}}=s_{0}+\frac{u_{q\_\mathrm{opt}l}}{L_{s}}\\ s_{j}={\frac{di_{q}}{dt}|}_{u_{q}=u_{\mathrm{qj}}}=s_{0}+\frac{u_{\mathrm{qj}}}{L_{s}} \end{array}$$ (11)

By substituting *u*_d_, *u*_q_ from Eq (8) into the prediction model of Eq (6), the predicted current of the second optimal voltage vector *V*_opt2_ can be obtained as follows:

$$\begin{array}{l} i_{d}^{j}(k+1)=i_{d}(k)+\frac{T_{s}}{L_{s}}[u_{d}{|}_{k=j}‐R_{s}i_{d}(k)+\omega_{r}(k)L_{s}i_{q}(k)]\\ i_{q}^{j}(k+1)=i_{q}(k)+\frac{T_{s}}{L_{s}}[{u_{q}|}_{k=j}‐R_{s}i_{q}(k)‐\omega_{r}(k)L_{s}i_{d}(k)‐\omega_{r}(k)\psi_{f}] \end{array}$$ (12)

By substituting the predicted current $i_{d}^{j}(k+1),i_{q}^{j}(k+1)$ obtained using Eq (12) into the value function of Eq (7) for comparison, *V*_opt2_ and the action time of the two voltage vectors can be obtained.

Thus, the action time and the optimal voltage vector of the q-axis current deadbeat can be obtained using the procedure described above. By replace the q-axis current in Eq (9) with the d-axis current in Eq (11) and repeating the above procedure, the d-axis current can be calculated. The action time under the difference shot and the optimal voltage vector respectively is substituted into the value function for comparison, and the optimal outcome is selected as *V*_opt2_.

**Fig 6:**
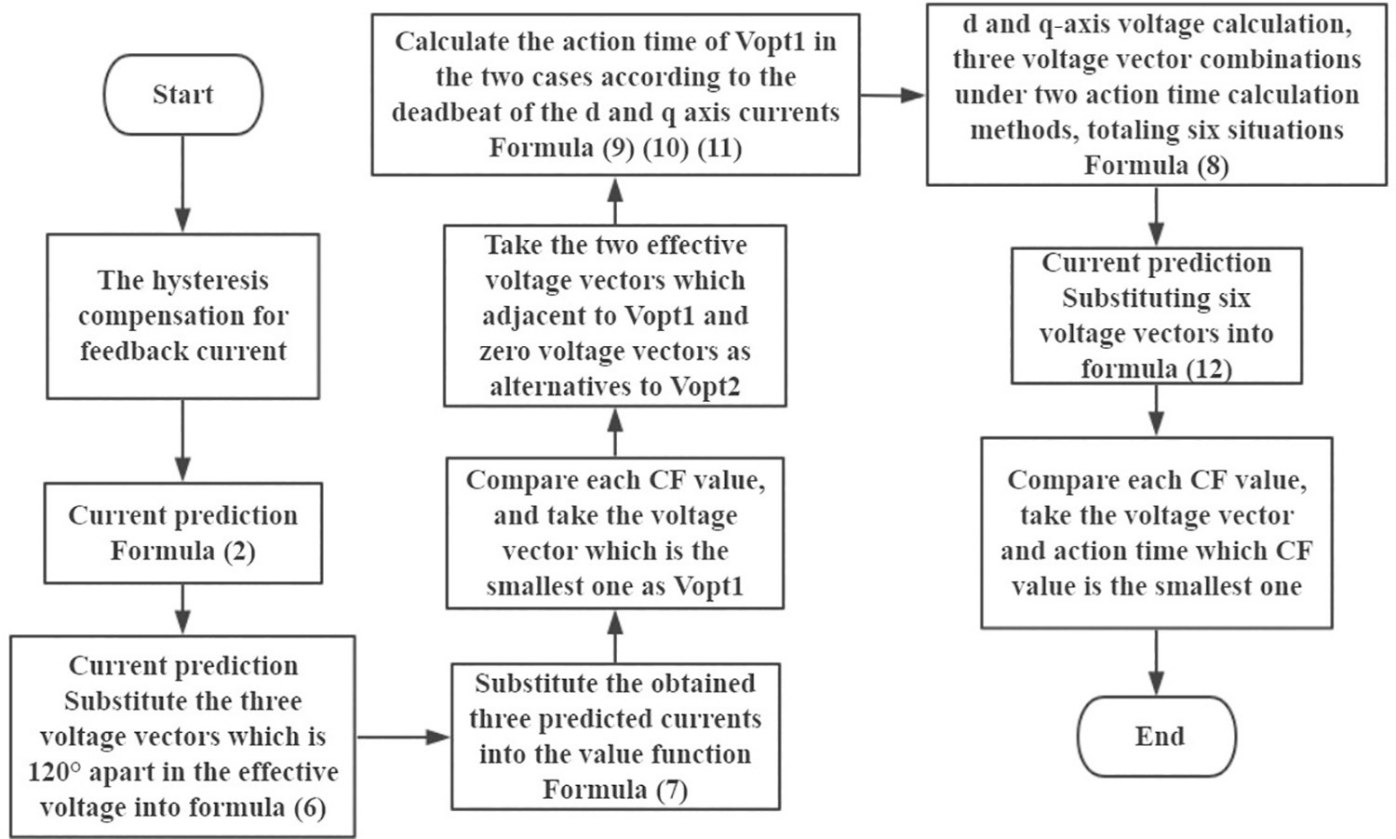
Flowchart of the dual-vector MPCC strategy.

The flow chart of the improved dual-vector MPCC method is shown in Fig 6.

## 4 Results and discussion

### 4.1 Results

In order to verify the feasibility of the proposed control algorithm, a simulation model was built in the MATLAB/SIMULINK environment. The main parameters of the motor in the simulation are listed in Table 1. In addition, in order to verify the effectiveness and superiority of the parameter algorithm, the traditional vector control simulation model and the traditional MPCC simulation model were built for the purpose of comparison under the same simulation conditions.

**Table 1:** Main parameters of PMSM used in the simulation.

d-axis inductance (mH)	8.5
q-axis inductance (mH)	8.5
Winding resistance per phase (Ω)	0.135
d-axis permanent magnet flux (Wb)	0.1827
Number of pole pairs	20
Rotor inertia (kg·m^2^)	0.027
Viscous damping (N·m·s)	0.0004924
Rated speed (r/min)	1000
Rated torque (N·m)	20
DC voltage (V)	540
frequency (Hz)	10^6^
Current proportional and integral coefficient	0.5,25

Three control strategies were simulated under different working conditions. First, the motor starts running at a constant speed of 1000 r/min and no-load torque. Then, the motor is loaded with a constant load torque of 20 N·m at 0.1 s. Finally, at 0.3 s, the given speed changes suddenly, and the speed is changed from 1000 r/min to 1500 r/min. The steady-state simulation experiment was carried out under the above conditions.

**Fig 7:**
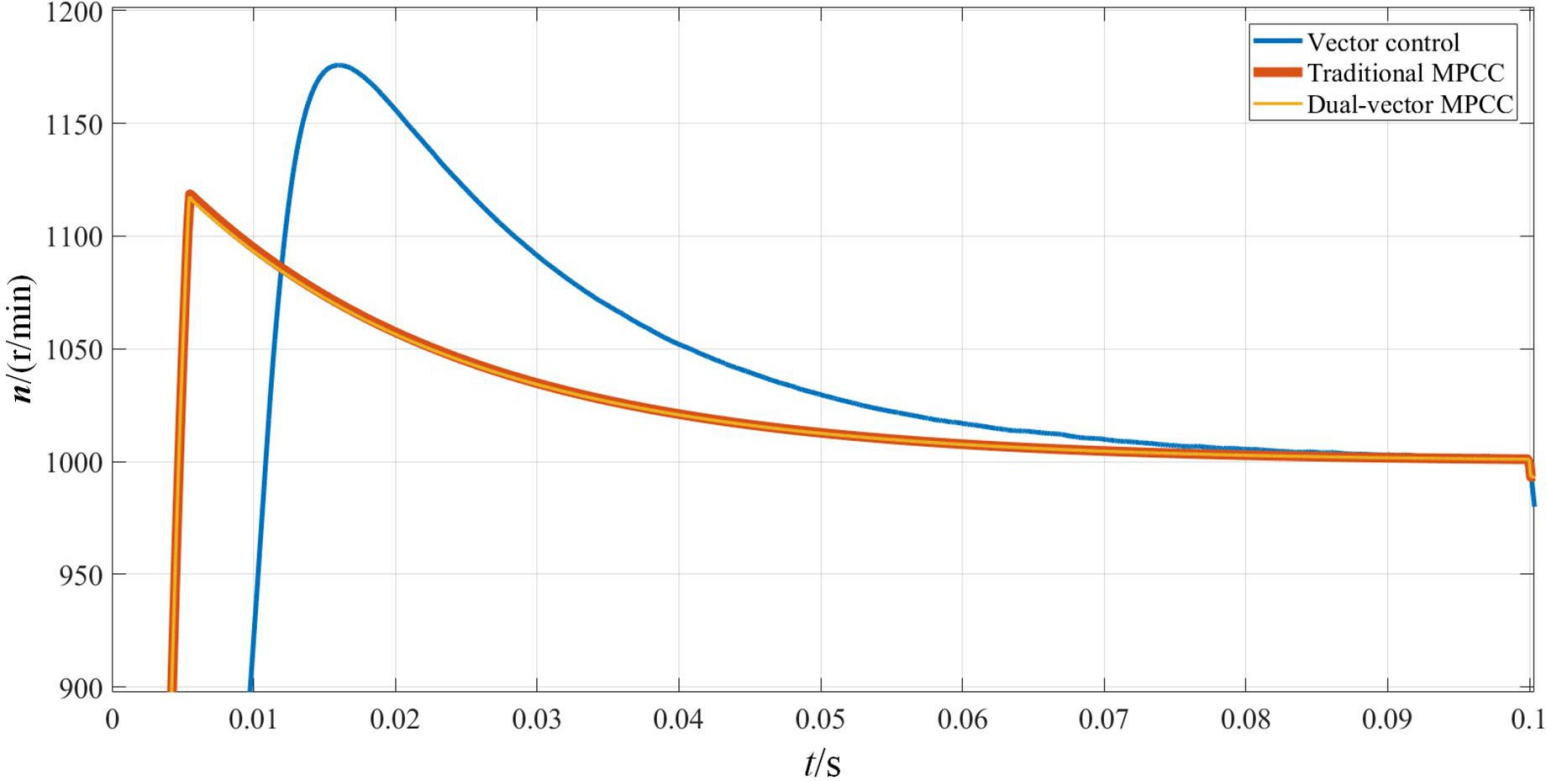
Rotational speed waveform when the motor starts.

**Fig 8:**
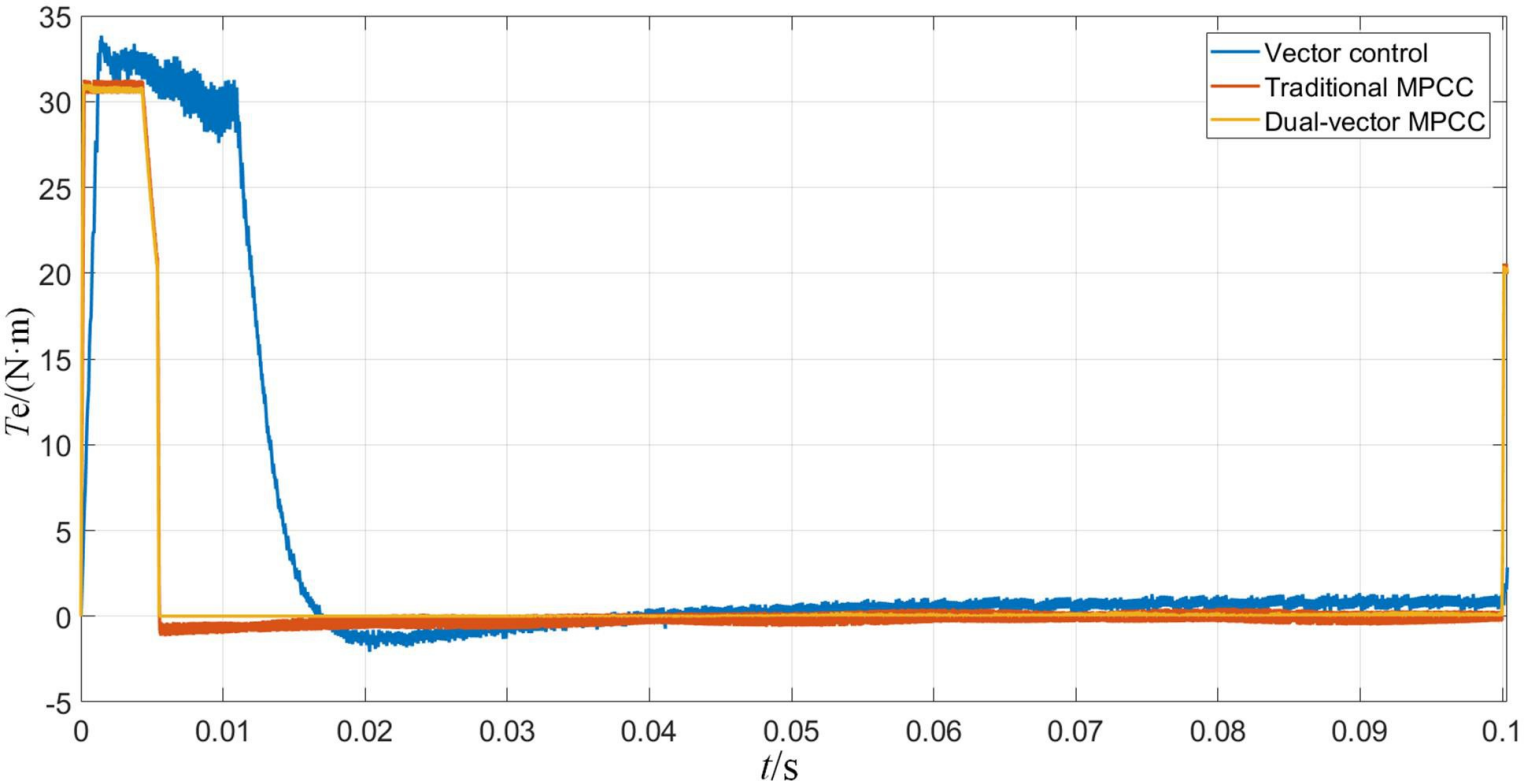
Torque waveform when the motor starts.

**Fig 9:**
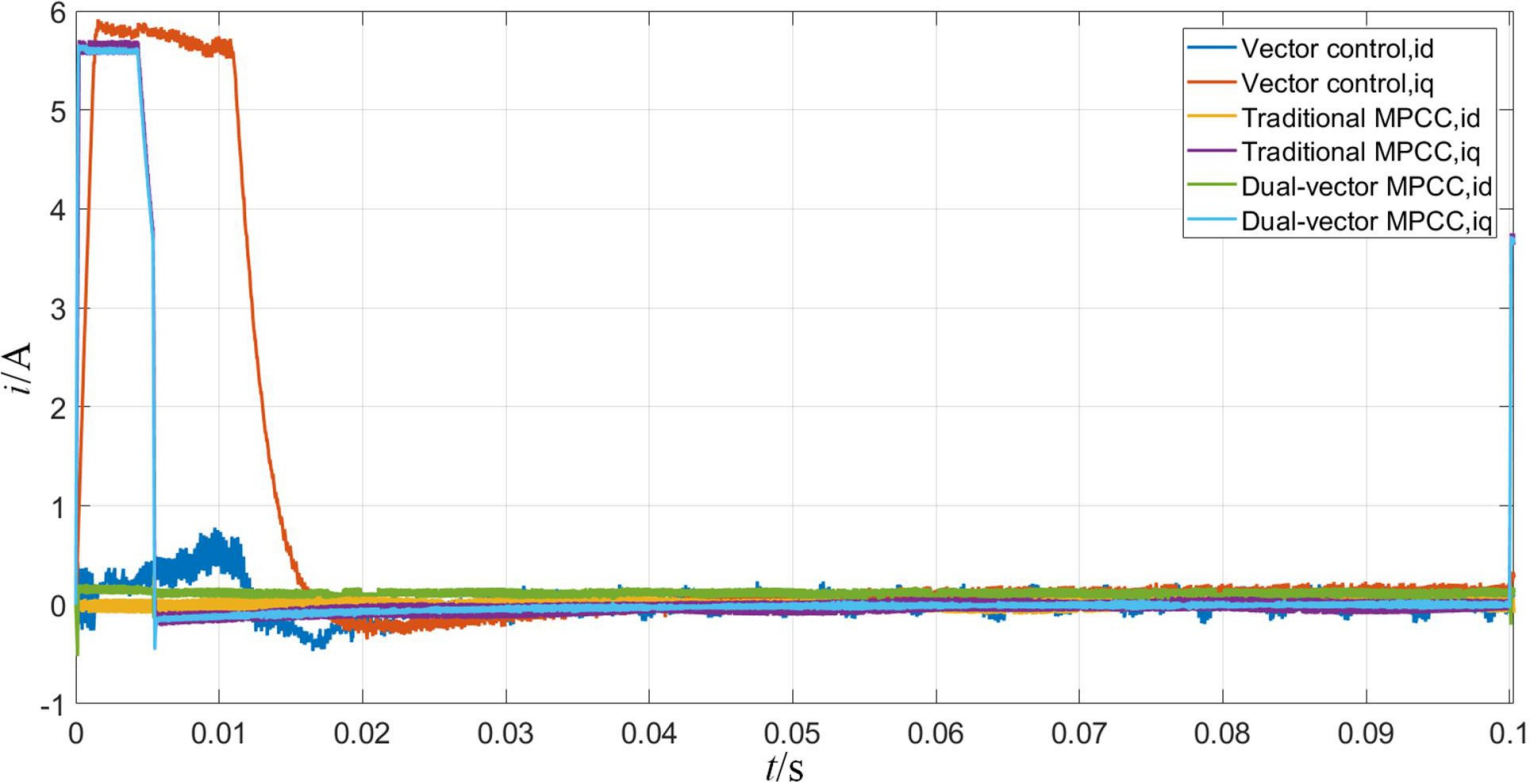
d-q axis current waveform when the motor starts.

Figs 7–9 shows the waveforms obtained when the motor starts under the three control strategies. As is observed from Fig 7, when the motor starts at no load, the three control algorithms can obey the given motor speed. Among them, the MPC strategy exhibits a smaller overshoot compared to the vector control strategy and can obey the given speed faster. As can be seen from Fig 8, the motor torque is stabilized at 0 N·m faster because of the MPC, and the time used is approximately 0.005 s, whereas the torque achieved using the vector control strategy requires 0.02 s to reach a stable state. As can be seen from Fig 9, the d-axis current stabilizes at approximately 0.1 A, whereas the q-axis current remains at approximately 5.8 A for a period of time and then decreases to the same level as the d-axis current. The shaft currents corresponding to both axes are similar, whereas the MPC maintains a shorter time and faster recovery.

**Fig 10:**
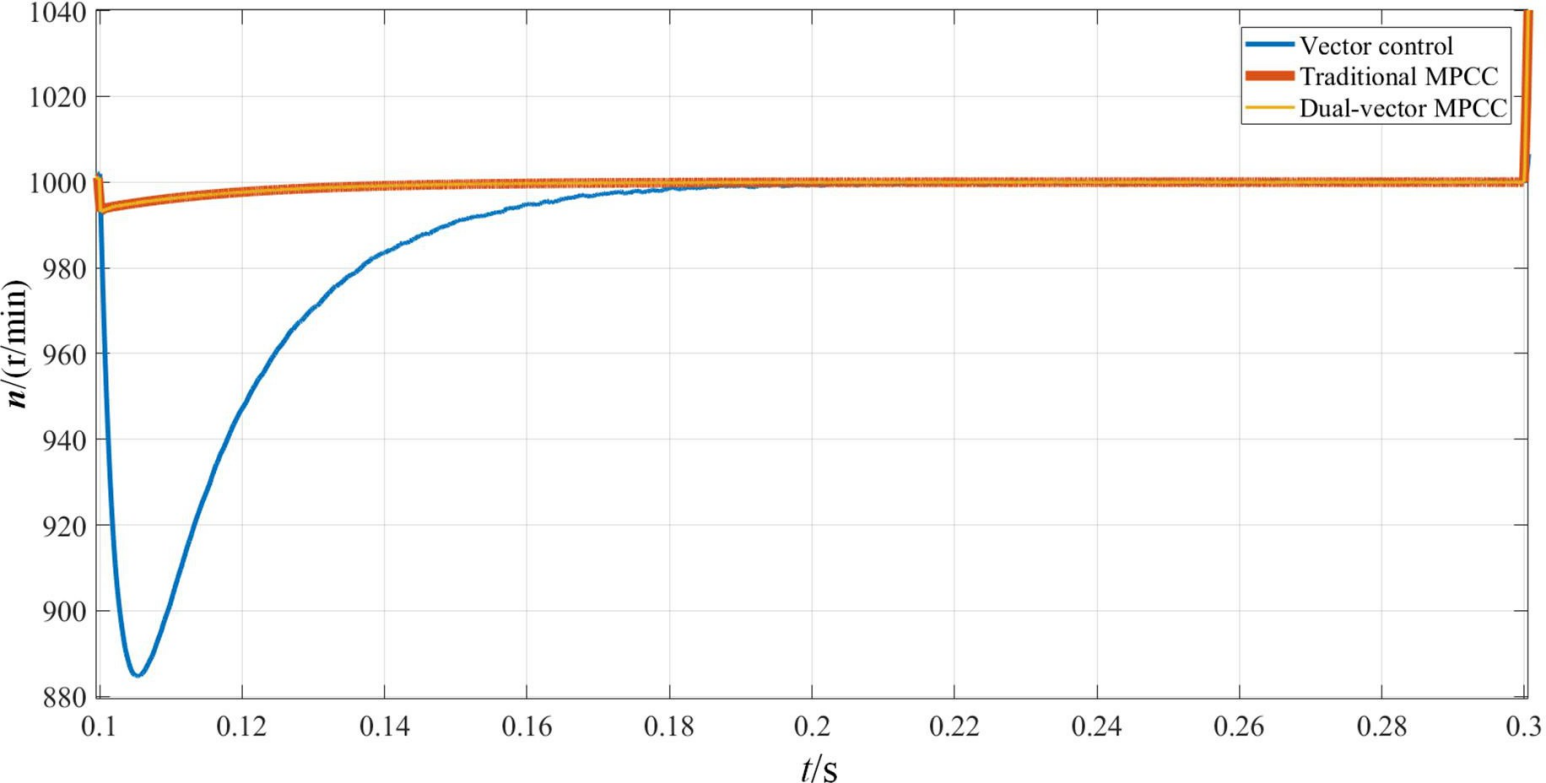
Rotational speed waveform when the motor is loaded with torque.

**Fig 11:**
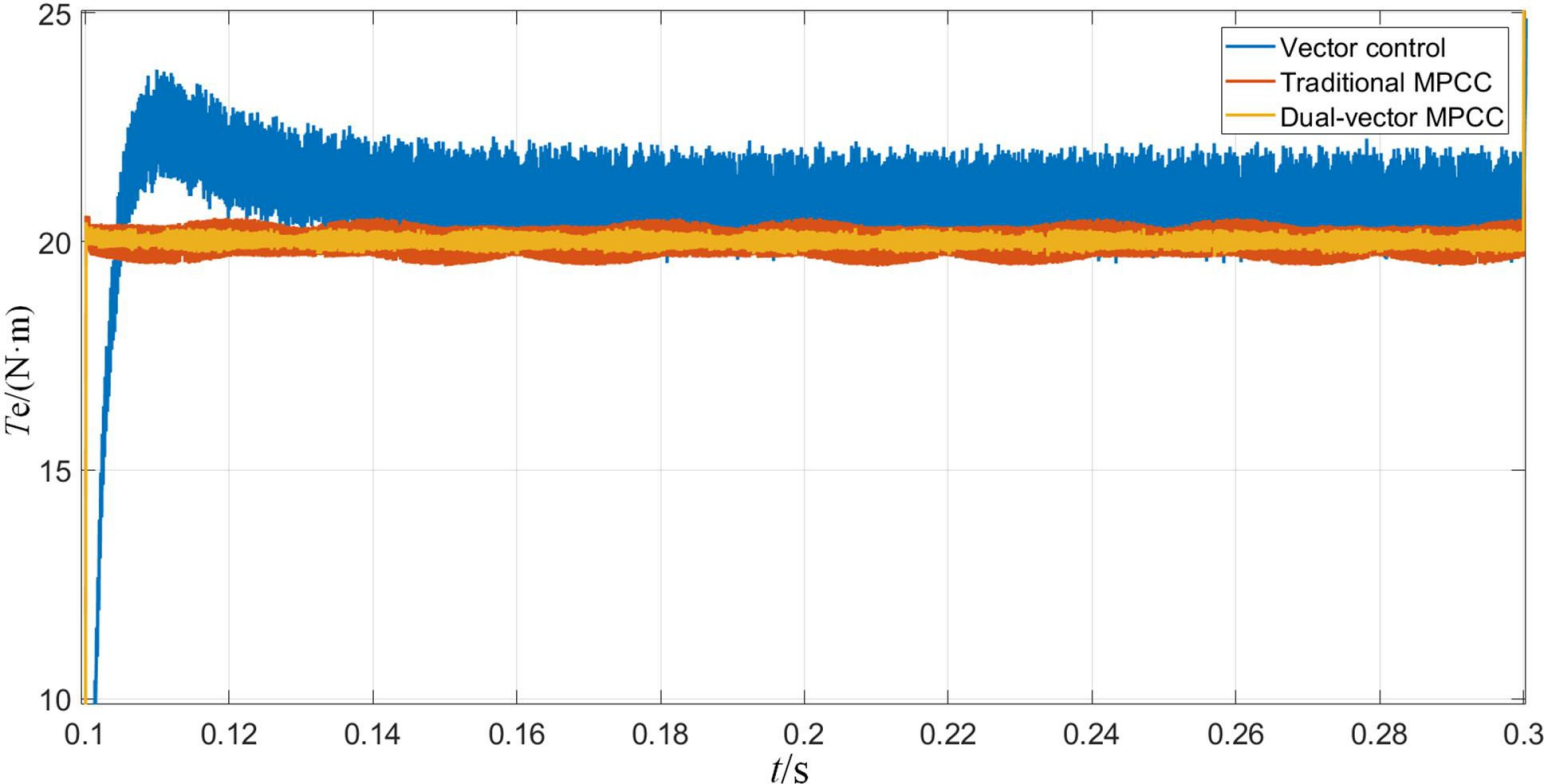
Torque waveform when the motor is loaded with torque.

**Fig 12:**
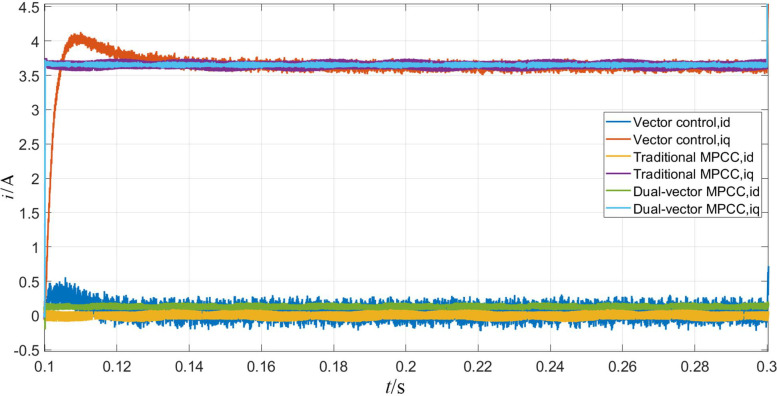
D-q axis current waveform when the motor is loaded with torque.

The simulation results corresponding to the sudden application of load torque to the PMSM are shown in Figs 10–12. In terms of speed, as shown in Fig 10, the speed under vector control suddenly drops to about 890 r/min and returns to a input speed in about 0.1 s. In contrast, the speed under MPC will be affected very slightly. It reduces by less than 10 r/min and returns to the given value within 0.05 s, and obviously has a stronger anti-interference ability. As can be seen from Fig 11, the MPC strategy enables the motor to achieve a faster and stable torque. On the other hand, the torque under vector control produces a large overshoot, which reaches stability after 0.04 s. As is observed from Fig 12, the d-axis current remains unchanged, and the q-axis current increases to approximately 3.6 A. The dual-vector model predicts the smallest waveform pulsation under current control and exhibits better stability.

**Fig 13:**
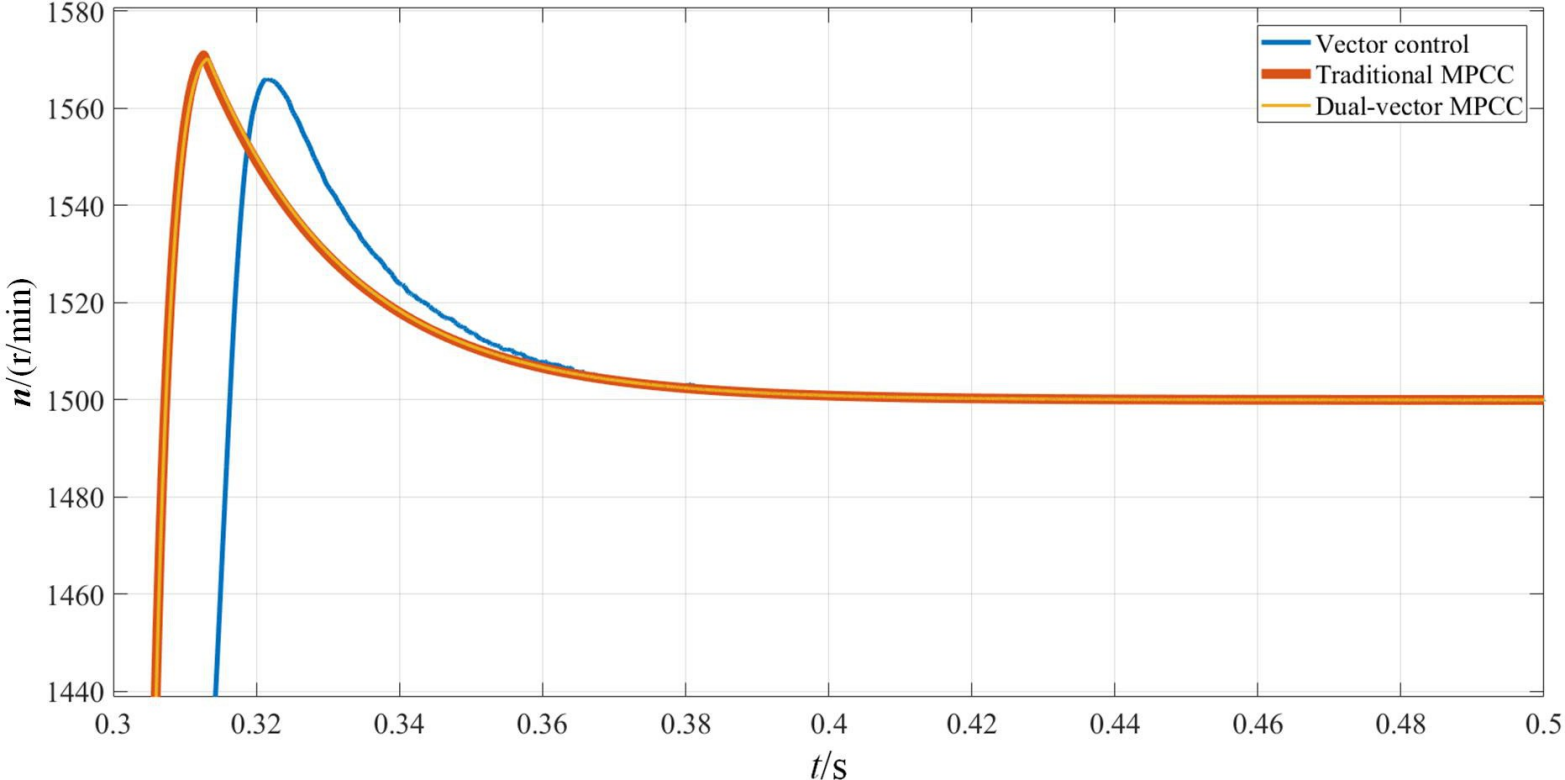
Rotational speed waveform when the given speed changes.

**Fig 14:**
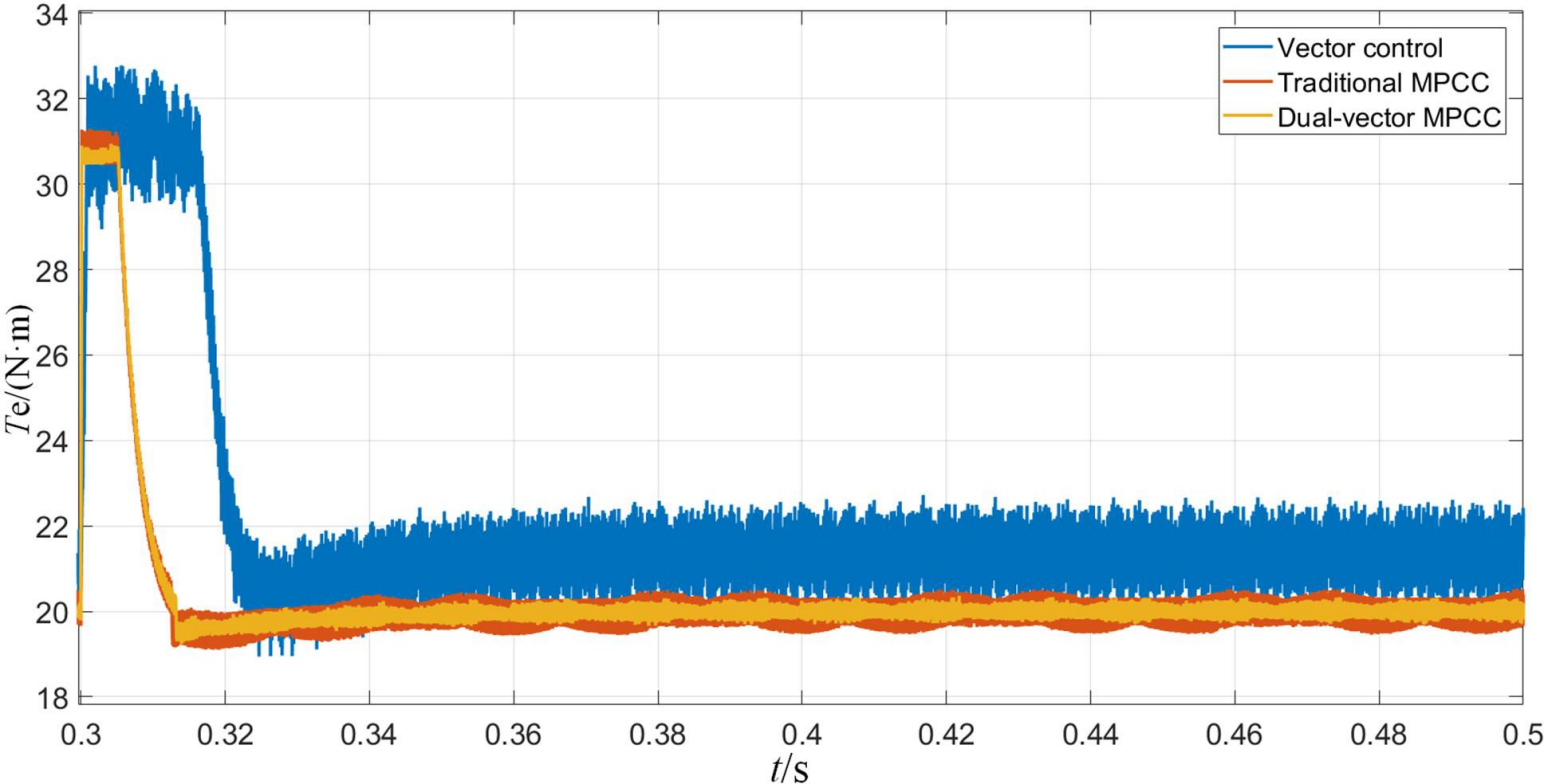
Torque waveform when the given speed changes.

**Fig 15:**
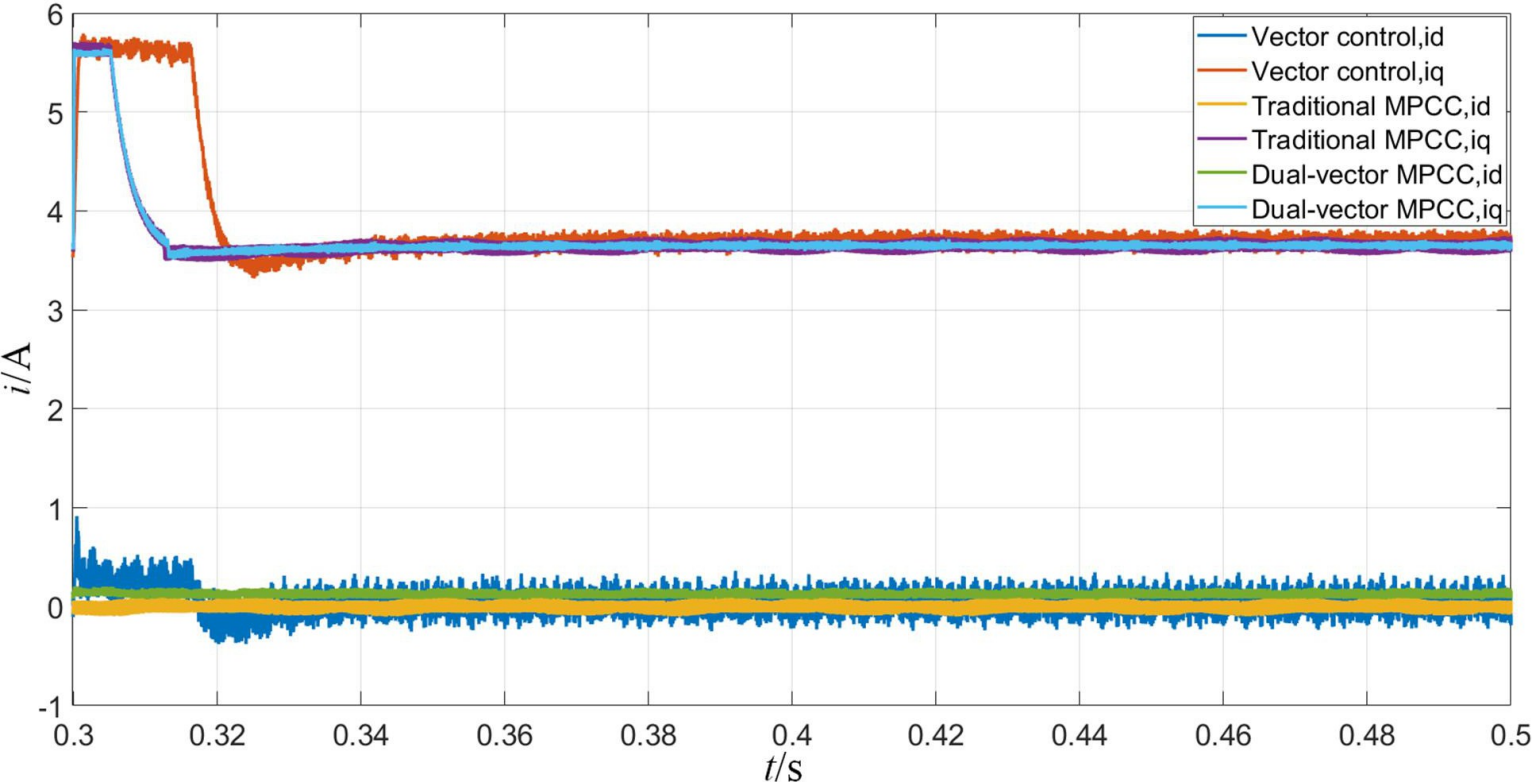
d-q axis current waveform when the given speed changes.

Figs 13–15 shows the experimental waveforms of the motor when the given speed of the control system changes. From Fig 13, it can be seen that the MPC provides fast speed switching. As can be seen from Fig 14, after the torque in the MPC system changes to 31 N·m, it returns to the load torque value of 20 N·m after about 0.01 s. On the other hand, in the vector control system, the torque is restored to the load torque value of 20 N·m. The moment value is increased to 32 N·m and maintained at 0.025 s. In contrast, the model predicts that the current control system has a better anti-interference ability. The d-and q-axis currents are shown in Fig 15, in which the q-axis current with a larger step is produced, whereas the d-axis current has a smaller step. Thus, from the results shown in the figure, it can be seen that the dual-vector model predicts that the current null value has smaller current fluctuations, its running state is stable, and its stability is better than the methods.

### 4.2 Discussion

Table 2 presents the results of the AC-DC axis current fluctuations of the three control strategies under different working conditions. From the table, it can be seen that when PMSM operates under the three operating conditions, the traditional MPCC and the dual-vector MPCC strategies can reduce the current fluctuations in the traditional MTPA. Comparison of the stator current obtained from the dual-vector MPCC to the difference between the d- and q-axis components of the traditional MPCC stator current shows that the fluctuations were obviously reduced, and better steady-state performance is obtained owing to the former method.

**Table 2:** Average current ripple of the three control strategies under different working conditions.

	Time	Δ*i*_d_/A	Δ*i*_q_/A
Vector control	start up (0.05–0.1s)	0.482	0.27
	Add load torque (0.15–0.25s)	0.563	0.281
	Change speed (0.35–0.45s)	0.632	0.294
Traditional MPCC	start up (0.05–0.1s)	0.185	0.264
	Add load torque (0.15–0.25s)	0.186	0.191
	Change speed (0.35–0.45s)	0.192	0.192
Two-vector MPCC	start up (0.05–0.1s)	0.147	0.102
	Add load torque (0.15–0.25s)	0.143	0.094
	Change speed (0.35–0.45s)	0.141	0.103

From the comparison of the simulation results presented above, it can be seen that the dual-vector MPCC strategy exhibits the same rapid torque and speed response as the traditional MPC strategy, and on this basis, the dual-vector MPCC strategy significantly reduces the current fluctuation in the traditional MPCC strategy. This is because, from the perspective of the voltage vector selection range, the traditional MPCC can only choose from seven voltage vectors, whereas the voltage vector selection range of the dual-vector MPCC is more extensive, and the direction of the two optimal voltage vectors is arbitrary. Voltage vector with adjustable amplitude. From the perspective of the accuracy of voltage vector selection, it has been found that in the traditional MPCC method, only the voltage vector whose predicted value is the closest to the given value is selected, which in principle cannot meet the current deadbeat requirement. However, in the dual-vector MPCC strategy, the second voltage vector selection can make the selected voltage vector more accurate by selecting a non-zero voltage vector in order to achieve the current deadbeat requirement in more sampling periods. Therefore, the dual-vector MPCC strategy can select the voltage vector in a larger vector selection range, which makes the selection of the voltage vector more accurate, achieves better steady-state performance, and greatly improves the robustness of the control system.

## 5 Conclusion

This study aims to address the problems of poor anti-interference ability and large torque fluctuations in the traditional vector control strategies. For the same, the traditional motor control strategy has been analyzed in this study in combination with the MPC theory for a system at a given torque or external load torque. Under the conditions such as sudden changes in the torque and the AC-DC axis current coupling enhancement when the motor is running at high speed, the current controller is saturated, and the actual working speed of the motor cannot be effectively tracked. Thus, an improved control method has been proposed in this study, in which the dual-vector MPCC strategy has been adopted, and the voltage vector selection is performed twice in each sampling period. In this strategy, the voltage vector is selected in a larger vector selection range to enable an accurate selection of the voltage vector and achieve good steady-state performance. When selecting the second voltage vector, the selection object is simplified, the number of calculations is reduced, the operating efficiency is improved, and the original 14 voltage comparisons are reduced to six. The simulation results show that the proposed control strategy not only inherits the performance of the traditional vector control strategy efficiently but also provides considerable robustness to system disturbances, thus greatly improving the anti-interference ability of the system.
